# Depiction of Physiological Homeostasis by Self-Coupled System and Its Significance

**DOI:** 10.3389/fphys.2019.01205

**Published:** 2019-09-19

**Authors:** Xia Lu, Guantao Jin, Wenjin Chen, Xinguang Yu, Feng Ling

**Affiliations:** ^1^Department of Neurosurgery, Chinese PLA General Hospital and Medical School of Chinese PLA, Beijing, China; ^2^Department of Neurosurgery, Xuanwu Hospital Capital Medical University and China International Institution of Neuroscience, Beijing, China; ^3^Advanced School of Art and Humanities, Chinese Academy of Art, Hangzhou, China

**Keywords:** self-coupled system, negative feedback system, homeostasis maintenance, continuous monitoring, weakened regulatory function induced disease

## Abstract

The negative feedback system (NFS) was regarded as the basic unit of regulation of physiological homeostasis for more than 70 years. However, NFS-based depiction possesses some limitations. The self-coupled system (SCS), a non-stop system in which the output of the current moment becomes the input of the next moment, can also be utilized to depict homeostasis. In SCS-based depiction, all of the related regulatory mechanisms of a homeostasis are regarded as an entity. Then, homeostatic dynamics can be expressed by simple mathematical language. A new disease group was revealed and some useful inferences were obtained through mathematical deduction. They were supported by published studies. SCS-based depiction of homeostasis should be a requisite supplement to medical knowledge systems based on NFS.

## Introduction

Since being scientifically described by Norbert Wiener, the negative feedback system (NFS) has been regarded as the basic modality of physiological homeostasis (Wiener, 1961). In such knowledge framework, the causality of mechanisms underlying homeostatic maintenance is linear, and functions of different structures and molecules can be seated accurately. Thus, in medical practice, therapeutic targets of diseases can be determined. However, depicting homeostasis with NFS still possesses some limitations.

First, to understand an NFS, one must know its sensor, regulating center, effector, and set-point. Not every homeostasis, however, has a perfect regulating center and a typical set-point, such as body temperature (Boulant, 2006). In numerous scenarios, clinicians only want to know the anti-jamming capability of a system. For example, will blood pressure increase dramatically after drinking, or fall seriously after anesthesia induction? Will glycemic fluctuate vigorously after diet or insulin injection? Most of the time, it is impossible for a doctor to determine the status all of the components of an NFS to evaluate its anti-jamming capability.

Second, it is inconvenient to express NFS by a mathematical method. The mathematical method used by Wiener is too complicated for most clinicians to understand, and even more difficult to use.

Third, sometimes it is confusing to use NFS to understand human homeostatic dynamics, which links health and disease. For example, a stable value of blood pressure (BP) fluctuates in a day (Parati et al., 2013). For a healthy person, all of these values are normal. It is thus confusing to depict these normal BP values with the term “set-point.”

Regarding all of the regulatory mechanisms as an entity will be helpful to overcome those limitations. Such recognizing modality has been intentionally advocated and applied in modern medicine, e.g., as the regulatory function of cerebral blood flow (CBF) (Aaslid et al., 1989; Eide et al., 2012; Willie et al., 2014). Such cognitive paradigm is called “systems theory” in scientific philosophy (Bertalanffy, 1950), and differs from reductionism. The theoretical basis underneath the systemic method in modern medicine is rarely understood. We will introduce the self-coupled system (SCS) to depict homeostasis, and thus establish a unified systemic perspective for modern medicine. We also believe that this is a requisite supplement to NFS-based knowledge systems.

## Rationality of Scs as the Basic Unit for the Depiction of Human Homeostasis

### Definition of SCS

**FIGURE 1:**
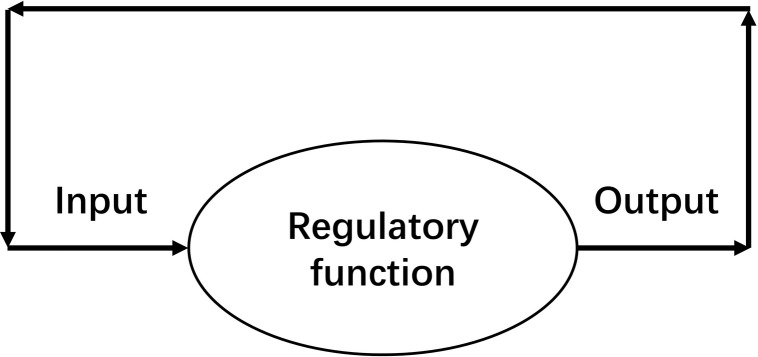
Schematic diagram of a self-coupled system (SCS), which is a system in which its output of this moment becomes the input of the next moment. A non-stop working SCS can maintain a homeostasis.

An SCS is defined as “a non-stop system in which its output of this moment becomes the input of the next moment” (Figure 1). In SCS, homeostasis is maintained by iteration of regulatory function. The attractor of an SCS is the stable value of a homeostasis.

### The Approximate Stable Value of a SCS

Define *x*_t_ as the value at time *t*, and *f*(*x*) as the expression of regulatory function. Define the duration required for finishing a regulatory action as a time unit. Then, an SCS must have:

(2.1) $$x_{t+1}=f\,\left(x_{t}\right)$$

The stable value is the fixed point or attractor of Eq. 2.1. When defining *x*_*0*_ as a stable value, there is:

$$x_{0}=f\,\left(x_{0}\right)$$

For determination of the approximate stable value of *x*_*0*_, Taylor series expansion is performed near the zero point:

$$x_{0}=f\,\left(x_{0}\right)=f\,\left(0\right)+\frac{f^{\prime }\,\left(0\right)}{1!}\,x_{0}+\frac{f^{"}\,\left(0\right)}{2!}\,x_{0}^{2}+\ldots$$

Neglect the high-order terms:

$$x_{0}=f\,\left(0\right)+\frac{f^{\prime }\,\left(0\right)}{1!}\,x$$

Make*f*(0) = c, *f*′(0) = *k*. Then, solving the equation, we obtain:

(2.2) x0=c(1−k)

So, c(1−k) is the approximate stable value of an SCS.

### Maintenance of Homeostasis

When deviation occurs and the status value changes from *x*_*0*_ into *x*_*t*_ at time *t*, for *x*_*t*_, there is:

$$x_{t+1}=f\,\left(x_{t}\right)$$

By performing Taylor series expansion near *x*_*0*_, then:

$$f\,\left(x_{t}\right)=f\,\left(x_{0}\right)+\frac{f^{\prime }\,\left(x_{0}\right)}{1!}\,\left(x_{t}-x_{0}\right)+\frac{f^{"}\,\left(x_{0}\right)}{2!}\,{\left(x_{t}-x_{0}\right)}^{2}+\cdots$$

When the deviation is small, the high-order terms can be neglected. Then:

$$f\,\left(x_{t}\right)=f\,\left(x_{0}\right)+f^{\prime }\,\left(x_{0}\right)\,\left(x_{t}-x_{0}\right)$$

Make *f*′(*x*_0_) = *k*, as *x*0 = *f (x*_0_) and *x*_*t* + 1_ = *f* (*x*_*t*_). So:

$$x_{t+1}-x_{0}=k\,\left(x_{t}-x_{0}\right)$$

Similarly, from *x*_*t+2*_ to *x*_*t+n*_, there will be:

$$x_{t+2}-x_{0}=k\,\left(x_{t+1}-x_{0}\right)=k^{2}\,\left(x_{t}-x_{0}\right)\,\ldots$$

(2.3) $$x_{t+n}-x_{0}=k^{n}\,\left(x_{t}-x_{0}\right)$$

Equation 2.3 shows how deviation was corrected by iteration of regulatory function.

By use of the method of self-coupled analysis, it has been proven that, despite that Eq. 2.3 was obtained near the stable value, it remains reasonable to use this equation to approximatively express all deviation corrections (Jin et al., 2019; Supplementary Data Sheet S1). The deductions from Eq. 2.3 are still tenable when *k* is not constant, but keeps |*k*| < 1, which is the situation of physiological homeostasis with a single stable value. And such stable value was termed “strange attractor” by Hofstadter (1981). In other words, for analysis of most physiological homeostases, *f*(*x*) can be seen as a linear function. To simplify the analysis, the following deduction was based on a constant *k*.

According to Eq. 2.3, if |*k*| < 1, irrespective of the value of *x*_*t*_, after a sufficiently long period, i.e., *n* is sufficiently large, there will always be *x*_*t* + *n*_−*x*_0_ = 0. That is the deviation of a system being corrected. For an SCS, maintenance of homeostasis needs |*k*| < 1. And the closer |*k*| approaches to *0*, the less time is needed for deviation correction, which means the stronger is the regulatory function.

Define *s*_*t*_ as deviation at time *t*,i.e., *s*_*t*_ = *x*_*t*_−*x*_0_. Then, Eq. 2.3 is changed into:

(2.4) $$s_{t+n}=k^{n}\,s_{t}$$

### Relationship Between NFS and SCS

An NFS can be regarded as an SCS whose input and output are the goal discrepancy, i.e., the difference between the status value and the set-point. Similarly, it can be expressed mathematically.

For an NFS, define the set-point as *G*, *x*_*t*_ as the status value at time *t*, and *f*(*x*) as the regulatory function of goal discrepancy. Also define the duration required for finishing a regulatory action as a time unit. The goal discrepancy at time *t* is *x*_*t*_−*G*. Then:

$$x_{t+1}-G=f\,\left(x_{t}-G\right)$$

Similar to the acquisition of Eq. 2.3, there will be:

$$x_{t+n}-G=k^{n}\,\left(x_{t}-G\right)$$

If |*k*| < 1 while *n*→ + ∞, always has:

$$x_{t+n}=G$$

This is the deviation corrected to the set-point.

Because NFS is a type of SCS, the conclusions drawn from the SCS-based deduction will not conflict against NFS-based knowledge.

### Classification of Regulation Patterns

**FIGURE 2:**
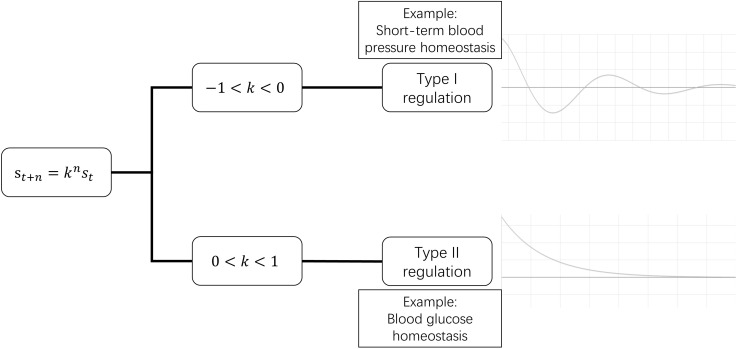
Two types of regulation patterns. In the time course of deviation correction, type I regulation exhibits fluctuating recovery, while type II regulation exhibits successive recovery.

According to Eq. 2.4, two types of regulation patterns are revealed (Figure 2):

Type I: When −1 < *k* < 0, s_t+n_/s_t+n–1_ has a plus-minus alternant sign. This means that, during the process of deviation being corrected, the status value is higher than the stable value in this moment, and lower in the next moment, i.e., the status value approaches the stable value in a similar way to a waning wave in a time-value curve.

Such pattern seems uneconomic, but it does exist in the human body. It can be seen in those homeostases whose deviations are corrected in a very short duration, such as very-short-term BP that is regulated by baroreflex (Dittmar and Mechelke, 1955; Eckberg, 1980; Rea and Eckberg, 1987) and neuronal membrane potential homeostasis (Hodgkin and Huxley, 1952).

Type II: When 0 < *k* < 1, always has s_t+n_/s_t+n–1_ > 0. So, during the process of deviation correction, the status value is always either higher or lower than the stable value. The status value progressively recovers to the stable value. Most clinicians are familiar with type II regulation.

### Shift of Stable Value and Occurrence of Disease

Disturbance to a homeostasis will lead to a shift of stable value. Disturbance changes regulatory function from *f*(*x*) to *F*(*x*), and the stable value was changed from *x*_*0*_ to *s*_*0*_. So, there is:

$$x_{0}=f\,\left(x_{0}\right)$$

$$s_{0}=F\,\left(s_{0}\right)$$

By use of self-coupled analysis, it was proven that when the structural stability of SCS was not damaged, the disturbed regulatory function can be expressed by *f*(*x*_*t*_) added with a linear item near *x*_*0*_ (Jin et al., 2019; Supplementary Data Sheet S1). In other words, when a Taylor series expansion is performed near *x*_*0*_ for *F*(*x*_*t*_), the result is adding *a*(*x*_*t*_−*x*_0_) + *b* to the expansion of *f*(*x*_*t*_), i.e.,

$$F\,\left(x_{t}\right)=f\,\left(x_{0}\right)+f^{\prime }\,\left(x_{0}\right)\,\left(x_{t}-x_{0}\right)+f^{{}^{\prime \prime }}\,\left(x_{0}\right)\,{\left(x_{t}-x_{0}\right)}^{2}\,\ldots$$

$$+a\,\left(x_{t}-x_{0}\right)+b$$

Neglect the higher items. Then:

$$F\,\left(x_{t}\right)=f\,\left(x_{0}\right)+f^{\prime }\,\left(x_{0}\right)\,\left(x_{t}-x_{0}\right)+a\,\left(x_{t}-x_{0}\right)+b$$

As *F*(*s*_0_) = *s*_0_,*f*(*x*_0_) = *x*_0_,*f*′(*x*_0_) = *k*,so:

$$s_{0}=x_{0}+k\,\left(s_{0}-x_{0}\right)+a\,\left(s_{0}-x_{0}\right)+b$$

(2.5) $$s_{0}-x_{0}=\overset{b}{\;}/\underset{\left[1-\left(k+a\right)\right]}{\;}$$

*s*_0_−*x*_0_ is the amplitude of the deviation of the new stable value compared with the primary one.

**FIGURE 3:**
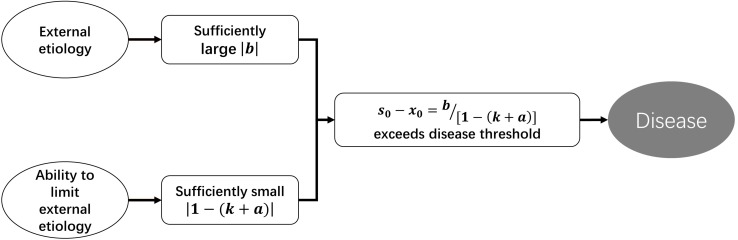
Pathogenesis under the perspective of SCS-depicting homeostatic dynamics.

According to Eq. 2.5, disturbance to a homeostasis can be divided into two categories. One targets the strength of regulatory function, *k* changed to *k+a*, and we term this “functional disturbance.” The other is *b*, which targets the status value directly without change of regulatory function, and we term this “direct disturbance.” According to Eq. 2.4, if *b*≠0, then *s*_0_−*x*_0_≠0, which means that deviation of homeostasis will exist persistently. Disease is deviation of homeostasis that exceeds a certain range. So, the occurrence of disease requires two conditions: |*b*| is sufficiently large, while |1−(*k* + *a*)| is sufficiently small. In fact, *b* represents external etiology (external to the homeostasis rather than the body), and 1−(*k* + *a*) represents the ability to limit the consequence of etiology. So, if the regulatory function of a homeostasis is weak enough, even insignificant external etiology can lead to disease (Figure 3).

Similarly, to simplify the analysis, the following deduction was based on a constant *k*, so that Eq. 2.4 can be utilized to analyze all stable value shifts.

### The Meanings of *k* Value

It has been proved when regarding all relevant regulatory mechanisms of a homeostasis as an entity, all physiological homeostases can be seen as SCSs. In the above deduction, *k* represents the inability of regulatory function, and its value reflects the type of regulation and the anti-jamming capability.

**FIGURE 4:**
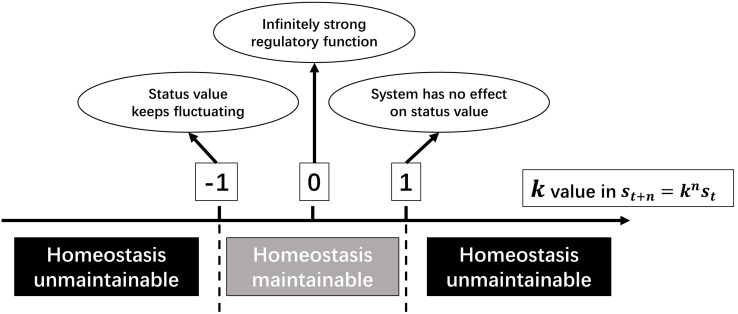
The meanings of *k* value in a SCS.

When |*k*| < 1, a homeostasis is maintainable. Moreover, the closer |*k*| approaches to 0, the stronger is the regulatory function; and the closer |*k*| is to 1, the weaker is the regulatory function. *k=0* means that the regulatory function is infinitely strong, which is impossible. When *k* = −1, the deviation fluctuates around the stable value with a fixed amplitude. Despite being difficult to imagine, there was still a proximate situation observed, i.e., baroreflex of a person with nervous disorders (Dittmar and Mechelke, 1955). When *k=1*, the regulatory system has no effect on the deviation. When |*k*| > 1, it is a positive feedback system, and the deviation will be amplified. If there is no regulation of a higher level, the homeostasis will collapse (Figure 4). As mentioned above, the sign of *k* value represents regulation patterns.

## Significance of Scs-Based Depiction of Homeostasis

### A Universal Methodology for Quantifying Regulatory Function of a Homeostasis

Regarding all of the regulatory functions of a homeostasis as entity is widely used in medical practice, usually by clinicians’ instincts. However, the methodology is often confused. SCS-based depiction provides a universal strategy to quantify the regulatory function, then quantifying clinicians’ instincts on anti-jamming ability becomes possible.

When the various physiological homeostases are examined in a highly admitted textbook of physiology (Hall, 2011) and by the “Tech-curves” window of the software HumMod (Hester et al., 2011), it will be found that most physiological curves are sigmoid, the segment near the stable value is approximatively linear, and no more than one attractor can be found by self-coupled analysis. In medical practice, quantifying the strength of regulatory function of this segment can satisfy most clinical demands, so the linear-based *k* value remains meaningful to evaluate the strength of the regulatory function of physiological homeostasis. Moreover, even the stable value shifts out of this segment as a result of disturbance, linear-approximation-based quantification is still meaningful, but clinicians must notice the result just represents the strength of that special period rather than the usual. Then the above equations can be utilized for not only qualitative, but also quantitative, analysis in medical practice.

Based on Eq. 2.4, the *k* value can be obtained by use of continuous monitoring data.

For type I regulation, continuous monitoring data will exhibit a wave-like time-value curve. According to *s*_*t* + 1_ = *ks*_*t*_, denote the initial deviation after a non-persistent interruption as *s*_*i*_, and the deviation of following first wave trough or wave peak as *s*_*t*_, then the *k* value can be acquired directly by:

(3.1) $$\overset{s_{t}}{\;}/\underset{s_{i}}{\;}=k$$

For type II regulation, the initial deviation was denoted as *s*_*i*_. After the duration of *t*, the deviation was changed to *s*_*t*_. During this process, the hypothetical number of times of regulation action *n=mt*, in which *m* is a constant index whose connotation is frequency in a time unit. Then:

$$s_{t}=k^{m\,t}\cdot s_{i}$$

As *s*_*t*_ and *s*_*i*_ have the same sign in type II regulation, natural logarithms can be taken bilaterally. Then:

$$l\,n\,s_{t}=m\,t\cdot l\,n\,k+l\,n\,s_{i}$$

$$\text{In}\left(\overset{s_{t}}{\;}/\underset{s_{i}}{\;}\right)=m\;.\;lnk\;.\;t$$

After giving a standard stimulation and measuring (*s*_*t*_,t) data groups, *m*⋅*lnk* can be worked out by linear regression. There is no need to know *s*_*i*_, which is impossible to be worked out in practice. Because *m* is a constant in a specific homeostasis, the relationship between *m*⋅ln*k* and *k* value is constant. So, the value of *m*⋅*lnk* can also be used as an indicator of the strength of regulatory function. We term *m*⋅*lnk* the “homeostatic index of type II regulation” and note it as:

(3.2) $$h=m\;.\;lnk=\overset{\text{In}\left(\overset{s_{t}}{\;}/\underset{s_{i}}{\;}\right)}{\;}/\underset{t}{\;}$$

According to numerous studies about blood glucose (BG) kinetics, as well as the familiar curves of glucose tolerance test (Ross and Tonks, 1938; Hamilton and Stein, 1942; Jokipii and Turpeinen, 1954; Lozner et al., 1956), the pattern of BG regulation is identified to be type II.

In the 1930s to 1950s, several studies attempted to find an empirical equation to mathematically depict the kinetics of excess BG elimination, which is called “glucose deviation correction” in our perspective. Some equations with satisfactory accuracy were introduced (Hamilton and Stein, 1942; Greville, 1943; Jokipii and Turpeinen, 1954; Silverstone et al., 1957). These equations have a similarity of exponential ones, and would give *K* values, i.e., the rate constant. Importantly, such *K* value is proven to be repeatable for individual (Elrick et al., 1956; Hlad and Elrick, 1959). Mathematically, all of the*K* values in those studies (Hamilton and Stein, 1942; Greville, 1943; Jokipii and Turpeinen, 1954; Elrick et al., 1956; Silverstone et al., 1957; Hlad and Elrick, 1959) have a fixed relationship with *h*.

For example, in the study conducted by Silverstone et al. (1957), their *K* value was worked out by:

$$k=\overset{ln\left(\overset{A}{\;}/\underset{y}{\;}\right)}{\;}/\underset{t}{\;}$$

where *y* is the BG concentration at time *t* following the injection of glucose load; and *A* is the theoretical maximum BG level at the time when mixing is complete (*t=0*). Obviously, in our deduction, *A* is the “initial deviation,” and also the *s*_*i*_ in Eq. 3.2. Then:

$$h=\overset{\text{In}\left(\overset{s_{t}}{\;}/\underset{s_{i}}{\;}\right)}{\;}/\underset{t}{\;}=\overset{\text{In}\left(\overset{y}{\;}/\underset{A}{\;}\right)}{\;}/\underset{t}{\;}=-K$$

Therefore, data in these studies also prove that the SCS-based quantifying regulatory function of glycemic homeostasis is both feasible and reliable.

### Revelation of a New Disease Group: Weakened Regulatory Function Induced Disease

For type II regulation (0 < *k* < 1), weakening of regulatory function (*k* gets closer to *1*) can be the only cause of disease. We term this “weakened regulatory function induced disease (WRFID).”

The stable value of homeostasis was denoted as *H*. According to Eq. 2.2, there is:

(3.3) $$H=\overset{c}{\;}/\underset{\left(1-k_{0}\right)}{\;}$$

As the numbers of stable values of physiological homeostases are usually positive, there must be c > 0. When c remains constant while *k*_*0*_ increases, *H* will increase. When the amplitude of this increase exceeds a certain range, WRFID occurs.

Moreover, it will be proven that even when c decreases, *H* can still increase.

Take differentials at both sides of Eq. 3.3. There will be:

(3.4) $$dH=\overset{\left[dc\;.\;\left(1-k_{0}\right)+dk_{0}\;.\;c\right]}{\;}/\underset{{\left(1-k_{0}\right)}^{2}}{\;}$$

For a linear function, Δ can be used to denote arbitrary change. Equation 3.4 is equivalent to:

(3.5) $$\Delta H=\overset{\left[\Delta c\;\;\left(1-k_{0}\right)+\Delta k_{0}\;.\;c\right]}{\;}/\underset{{\left(1-k_{0}\right)}^{2}}{\;}$$

In Eq. 3.5, even Δ*c* < 0,if 1−*k*_0_ is sufficiently small (*k*_*0*_ gets closer to *1*), Δ*H* > 0 can still occur. A Δ*H* that is too large leads to disease. Therefore, for a type II regulation (0 < *k* < 1) pattern, even without external etiology, like too much food intake to BG, weakening of regulatory function can still lead to disease, which must have a manifestation of higher status value. Importantly, for WRFID with very weak regulatory function, even though treatment can make c smaller, H can still be larger. In other words, for treatment of WRFID, improvement of regulatory function is critical.

### Two Provable WRFIDs

**FIGURE 5:**
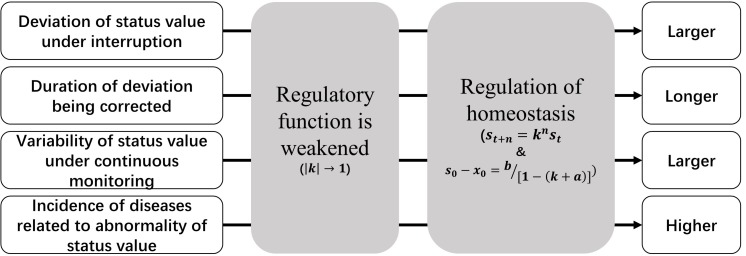
Manifestations of weakened regulatory function.

According to Eqs 2.4 and 2.5, the following inferences will exist for individuals with weakened regulatory function when compared with normal ones, as well as their healthy status (Figure 5):

(1) The same direct disturbance to status value will result in a larger deviation;
(2) The duration of deviation being corrected to the baseline will be longer;
(3) If the stable value is monitored continuously, the variability of the data will be larger;
(4) Such population will have a higher incidence of corresponded WRFID and diseases related to the higher or lower status value.

The above manifestations also constitute features of WRFIDs, and they will be helpful for identification of such diseases. Continuous monitoring of status values or their equivalent parameters is the precondition of identification. Indeed, there might be many WRFIDs. However, after extant literature review, we identified two WRFIDs that can be proved published data (Table 1).

**TABLE 1:** Summary of provable WRFIDs.

**Diseases**	**Type of evidence**			
	**Larger positive**	**Larger negative**	**Larger variability**	**Longer duration**
	**challenge resulting**	**challenge resulting**	**of continuous**	**of deviation being**
	**deviation**	**deviation**	**monitoring data**	**corrected to baseline**
T2DM	++	++	++	++
Primary hypertension	++	+	++	–

*++, direct evidence, i.e., there is a comparison between patients and control; +, indirect evidence, i.e., there is no comparison between patients and control, but there is evidence that shows that a too-low status value is a considerable problem of treated patients; –, lack of evidence. T2DM, type II diabetes mellitus.*

Type II diabetes mellitus (T2DM) is a provable WRFID. In the widely used glucose tolerance test, it is well-known that, in T2DM patients, the BG deviation results from a standard load will be higher and the duration of BG deviation corrected to the baseline is higher compared to healthy controls (Hall, 2011, pp. 951–953). Although there is no negative challenge test for BG, it is well accepted that the incidence of hypoglycemia is substantial high in treated T2DM patients. Moreover, it is demonstrated that the occurrence of hypoglycemia is related to a worse prognosis, which reflects a worse illness (Leese et al., 2003; Gore and McGuire, 2009; Seaquist et al., 2013). This phenomenon is in accordance with the predictions of WRFID. Continuous monitoring of BG showed that from healthy volunteers, to impaired glucose tolerance (IGT) persons, to T2DM patients, glycemic variability became increasingly large (Acciaroli et al., 2018). As variability reflects regulatory function, people with larger glycemic variability will have not only a higher incidence of hyperglycemia, but also hypoglycemia. This prediction has been proven in T2DM patients (Magri et al., 2018).

Primary hypertension is an another provable WRFID. Diagnosis of primary hypertension is based on the value of blood pressure measured by a proper technique after appropriate preparation, which includes sufficient rest and avoidance of factors influencing blood pressure level (Whelton et al., 2018). So, the related homeostasis of primary hypertension is the maintenance of short-term, mid-term, and long-term BP (Parati et al., 2013), which is type II regulation. This does not include the very short-term one, which is regulated by baroreflex (Parati et al., 2013), a type I regulation. Primary hypertensive patients have a higher BP reactivity to standard mental stress when compared to normotensive controls (Schulte et al., 1984), and even renal hypertension (Deter et al., 2007). In addition, exaggerated BP response to exercise, postural changes, loud noise, cold water immersion, and mental challenges were found to be predictors of future hypertension (Benbassat and Froom, 1986; Matthews et al., 1998; Miyai et al., 2000). Although no study yet exists that tests BP response to negative challenge, hypotension has become a considerable problem in treated hypertensive patients (Divison-Garrote et al., 2016; Wright et al., 2016). There is also evidence from continuously monitoring data. For example, a published study showed that the standard deviation (equivalent to variability) of mean arterial pressure in hypertensive subjects was greater than in normotensive subjects of similar ages (Mancia et al., 1980).

Non-WRFID diseases associated to weakening of a regulatory mechanism should be described by reductionism. The dynamic laws of them are simple. For example, when a mechanism functioning as lowering the status value was weakened, the stable value of homeostasis would be higher and a negative challenge would be hard to cause a major decrease.

## Discussion

Making use of SCS to depict human homeostasis provides a novel and useful perspective for medicine. However, SCS is will not replace NFS. SCS-based depiction of homeostasis should be a requisite supplement of NFS-based knowledge systems.

### Advantages and Limitations of SCS as a Basic Unit for Depiction of Human Homeostasis

Compared with NFS, SCS as a basic unit for the depiction of human homeostasis possesses the following advantages:

First, the mathematical expression is simpler. This method is easy to utilize, and we believe that most medical clinicians can understand it. After proving the rationality by self-coupled analysis, we used a linear function to express regulatory function, and obtained some valuable inferences. It is well-known that regulation of human homeostasis is so complex that a linear function can never match reality absolutely. However, the inferences expressed by natural language remain correct even when the *k* value is not constant, but keeps |*k*| < 1.

In medical practice, to quantify the regulatory function of a homeostasis of a period, the *k* value must be constant. However, the existence of homeostasis not suitable for linear approximation should be considered. Mathematical description with application value for non-linear scenarios will be studied in the future. The dynamics of *k* value is also meaningful in medical practice, especially for critical-ill patients, whose homeostatic status could change rapidly. The methodology dealing with a changing *k* value is to be developed.

Second, clinicians can evaluate the anti-jamming ability of a system by working out the *k* value or other equivalent parameters. If analyzing with NFS, clinicians must know the status of every component, including sensors, regulating centers, effector, and set-points, which is often impractical.

Third, homeostatic dynamics can be expressed by mathematical language easily. Equation 2.5 shows tersely how shifts of stable values occur. Such a simple expression makes clear sense, and further analysis assists us to reveal a disease group that has not yet been reported.

It is important to note that advantages in depiction do not always mean advantages in application. In SCS, all of the related regulatory mechanisms of a homeostasis are combined as an entity. When being used to guide therapy, SCS can just inform clinicians about strategy, rather than specific targets. This is the reason that we emphasize that such depiction is not a replacement, but a supplement, to the NFS-based one. Indeed, this constitutes a systems method, which must be combined with reductionism in treating disease.

### The Entity of Regulatory Function as a Therapeutic Target and Prognostic Factor

In the current paradigm of modern medicine, which is largely based on linear causality, it is very difficult to intervene in the entity of all of the related regulatory mechanisms at one time. However, some effective therapeutic or preventive management strategies do improve parameters that reflect regulatory functions.

Some studies exist that showed that, through some “easy” management, the incidence of WRFIDs may be decreased in susceptible populations, and corresponding regulatory functions of WRFIDs can be improved. For example, diet and/or exercise interventions have both preventive and therapeutic effects against T2DM (American Diabetes Association [ADA], 2018). Although the working mechanism remains unspecified, biguanide metformin is a widely used anti-hyperglycemic drug that improves BG control. However, it is not correlated with a higher incidence of hypoglycemia, suggesting improvement of BG regulatory function (Ferrannini, 2014). Another example of single anti-hyperglycemic drug improve the entity BG regulation is incretin-based drugs, which stimulates pancreatic beta cells as response only to high blood glucose but not hypoglycemia (Nauck et al., 2009). In addition to reducing BP in mild hypertensive patients, aerobic training may also have a favorable impact on BP reactivity of hypertensives during some stressful situations (Perkins et al., 1986). Aerobic exercise and physical activity were found to have effects of improving BP reactivity in young adult African-American women, who are prone to developing primary hypertension (Leary et al., 2000; Staffileno et al., 2007). Among various classes of antihypertensive drugs, calcium channel blocker (CCB) was also found to have the effect of lowering BPV, indicating improvement of BP regulatory function (Rothwell et al., 2010; Rakugi et al., 2015; Nozato et al., 2018).

Parameters that reflect regulatory function do predict prognosis in WRFIDs. For example, large glycemic variability has been well-accepted as a risk factor of diabetes complications (Hirsch, 2015). Many studies found that BPV, irrespective of being short-term or long-term, was a predictor of prognosis of hypertensives (Parati et al., 2013; Stevens et al., 2016). BPV was also thought to be a parameter of severity of illness for hypertension patients (Parati et al., 2013).

According to Eq. 2.5, diseases that do not belong to WRFIDs can also benefit from improvement of regulatory function. For diseases with etiologies that are unknown or impossible to eliminate, strengthening regulatory functions to minimize the damage of etiologies is a promising strategy. In other words, the period of living with diseases peacefully can be lengthened. There are also evidences supporting that physical activity or exercise can improve survival from colorectal, breast, and prostate cancers (Cormie et al., 2017; Friedenreich et al., 2017).

### The Usage of Personal Health Data From Wearable Devices

With developments in wearable devices, increasing numbers of human signs can be monitored continuously. Determining how to optimally use these tremendous data presents a problem. The most popular strategy at present is to seek the probabilistic dependencies between data patterns and body status. However, the reasons behind those probabilistic dependencies are frequently blurred. In other words, there is no theoretical support for those probabilistic dependencies. We assert that quantifying regulatory functions by the use of continuous monitoring data is a good strategy.

At last, we have to emphasize that all above hypotheses on application need to be tested by clinical trials.

## Conclusion

Self-coupled system is the more basic unit of human homeostasis, and SCS-based depiction is a requisite supplement to the NFS-based one. When using SCS to depict a homeostasis, all related regulatory mechanisms are regarded as an entity, and then simple mathematical language can be utilized to express homeostatic dynamics. After analyzing homeostatic dynamics by mathematical deduction, we found that, even without classical etiology, weakening of regulatory function can be the only cause of disease in type II regulation, which is termed “weakened regulatory function induced disease (WRFID).” Published data indicated that T2DM and primary hypertension are WRFIDs. For treatment of WRFID, improvement of regulatory function is critical. By use of continuous monitoring data, regulatory functions of homeostasis can be quantified to guide prevention and treatment, especially for multi-factor diseases. Compared with seeking probabilistic dependencies, SCS-based depiction provides a new and useful strategy for utilizing personal health data from wearable devices.

## Data Availability

The datasets generated for this study are available on request to the corresponding author.

## Author Contributions

FL conceptualized the research. GJ and XL developed the methodology. XL executed the research and wrote the initial draft under the supervision of GJ. WC provided part of the data and ideas. XY revised the manuscript.

## Conflict of Interest Statement

The authors declare that the research was conducted in the absence of any commercial or financial relationships that could be construed as a potential conflict of interest.
