# Supplementary material for: Depiction of Physiological Homeostasis by Self-Coupled System and Its Significance
Source: Front Physiol. 2019 Sep 19;10:1205. doi: 10.3389/fphys.2019.01205 (PMC6761279; doi:10.3389/fphys.2019.01205)
Supplement: Supplementary file 1 [file Data_Sheet_1.PDF]

Chapter 2

The Self-Coupling Analysis and the Structural Stability

Confucius said: "We do not know life, how can we know death?" Nowadays, perhaps it can be revised as "We do not know disease, how can we know life?"

Apparently, the true secret of life lies in the common knowledge that disease is the deviation from the normal values.

2.1 The Case of Blood Pressure Regulation

In order to introduce an analysis method that can be widely used in the study of pathology through combination of complex feedback mechanism in physiology and the structural stability of such regulatory mechanism, let us start with a case of blood pressure regulation.

Sec. 1.4 of Chapter 1 introduced ways that the long-term homeostasis of the mean arterial pressure (MAP) can be maintained. In fact, in order to maintain life, not only MAP needs to have long-term stability, the body must also respond quickly to external disturbances. For example, when the body has a greater degree of postural change (e.g. from supine to upright), there will be a short period of rapid fluctuations in blood pressure, nevertheless, the blood supply to the brain must be kept fairly stable, that is, MAP should still be maintained at around 100 mmHg (Fig. 2.1.1). In this case, besides maintaining the long-term stability of the general blood pressure, an acute regulatory mechanism to maintain a constant blood pressure in the brain is also necessary. Nowadays, this mechanism has been clarified, and is known as the arterial baroreceptor reflex (baroreflex). Its reflex arc is mainly composed of nervous centers, afferent and efferent nerves, as well as receptors and effectors in the cardiovascular system (Fig. 2.1.1). This mechanism is different from the way of maintaining MAP over a long period by regulating total intravascular blood amount (vascular filling). The overall regulatory process is as follows: the baroreceptors in the carotid sinus and aortic arch are monitoring changes of the pressure inside the carotid sinus and aortic arch at all time. When the pressure inside the carotid sinus and the aortic arch deviates from the normal values, the body will quickly adjust the cardiac output and the

世界图书出版西安有限公司

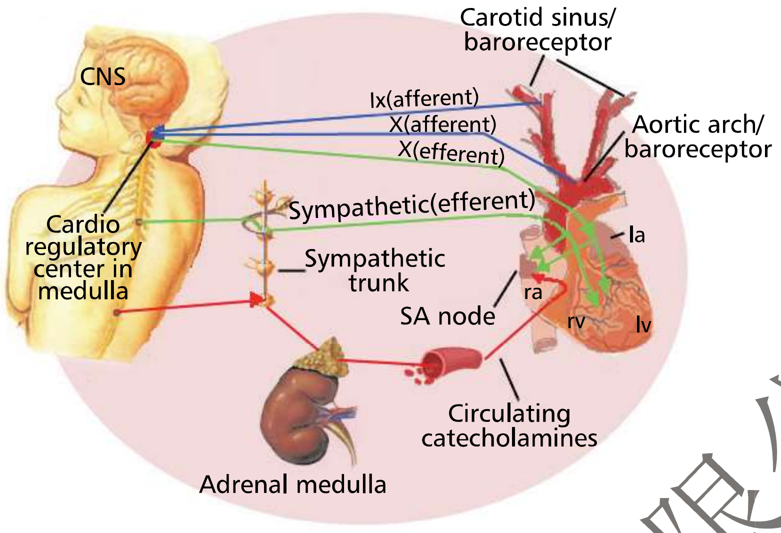

Fig. 2.1.1 The Arterial Baroreceptor Reflexes

peripheral vascular resistance to return the general blood pressure to the normal range.

According to a study by Dittmar et al., when the body posture was abruptly changed from supine to upright, the cardiac output quickly decreased in a short time due to a sudden drop of returned blood volume, which led to a sharp decline in blood pressure, activated baroreflex and caused the blood pressure to return to the normal range. In this process, changes in blood pressure are manifested in four types below (Fig. 2.1.2)[1]. The first and the third type occurred among normal subjects, while the second and the fourth type occurred among subjects with abnormal regulatory functions. Later on, these four manifested types will be expressed as logical functions.

Physiological studies have shown that there is a constant relationship between the carotid sinus pressure and the blood pressure post-regulation, which can be described as a sigmoid stimulus-response curve[2]. It was derived from the measurements of MAP or the heart rate corresponding to the changes of pressure inside the isolated carotid sinus [3]. As shown in Fig. 2.1.3, these changes are expressed as logic functions, where the x-axis represents the value of carotid sinus pressure, while the y-axis represents the value of MAP (also known as blood pressure) or heart rate responding quickly to the fixed carotid sinus pressure[4]. The currently known baroreflex curve equation was

[1] Silbernagl, Stefan and Despopoulos, Agamemnon, Color Atlas of physiology, Thieme, 2008. Original Data come from Dittmar, A., Mechelke, K., "Regulation of the Blood Pressure in Healthy Persons and in Nervous Cardiovascular Disorders", *Dtsch Arch Klin Med*, Vol.201(1955).

[2] Sagawa, K., "Baroreflex Control of Systemic Arterial Pressure and Vascular Bed", in Shepherd, J. T. and Abboud, F. M., ed., Handbook of physiology, The Cardiovascular System, American Physiological Society, 1983. Rowell, L.B., O'Leary, D.S., Kellog Jr., D.L., "Integration of Cardiovascular Control Systems in Dynamic Exercise", in Rowell, L.B., Shepherd, J. T., ed, Handbook of physiology: a Critical, Comprehensive Presentation of Physiological knowledge and concepts, Oxford University Press, 1996.

[3] Regarding the relationship between carotid sinus pressure and mean arterial pressure (MAP) currently attained, such research methodology always isolate the carotid sinus of the animals without damage to the innervation, then change the carotid sinus pressure manually and observe BP changes, finally get the fitting curve based on the multiple experimental results by regression. For a representative example, please refer to Sato, T., et al: "New Simple Methods for Isolating Baroreceptor Regions of Carotid Sinus and Aortic Depressor Nerves in Rats", *American Journal of Physiology*, Vol.276(1999).

[4] Kent, B.B., et al., "A Mathematical Model to Assess Changes in the Baroreceptor Reflex", Cardiology, V01.57(1972); Raven, P.B., et al., "Arterial Baroreflex Resetting During Exercise: a Current Perspective", *Exp Physiol*, Vol.91(2006).

世界图书出版西安有限公司

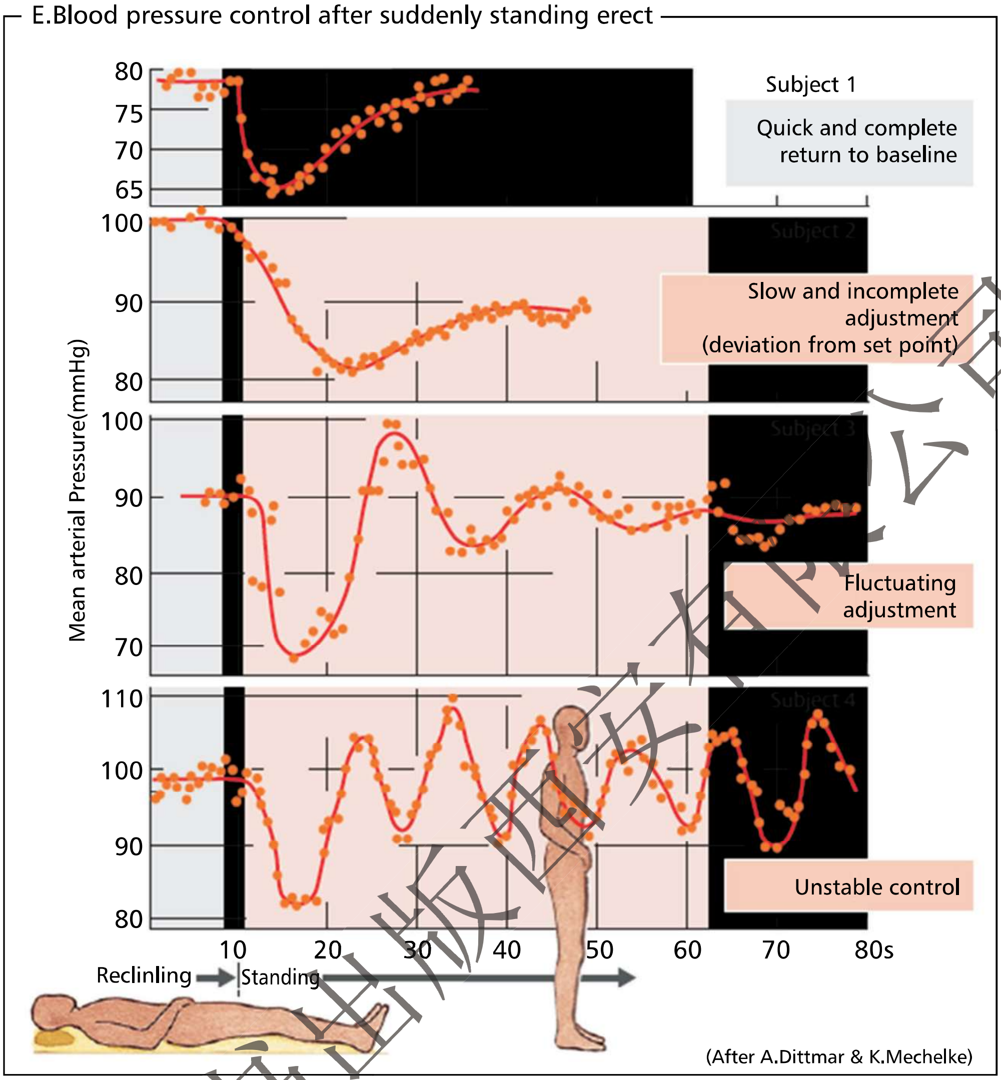

Fig. 2.1.2 The rapid fluctuations of BP when the posture is changed from supine to upright

proposed first by Kent et al, which can be expressed as:$y=A_4+A_1/[1+\exp\{A_2(x-A_3)\}]$. In the said figure, A_1 is the largest range of y; A_2 is the growth factor (reflecting the slope of the curve), A_3 is the central point; A_4 is the minimum average sinus pressure inducing the changes of MAP; exp (n) is a natural base e^n.

As shown in Fig. 2.1.3, when x is in the centering point A_3, $y=A_4+A_{1/2}$. Here, the centering point is exactly the midpoint within the responding range A_1. The line above the centering point represents pressor reflex, the line below the centering point represents depressor reflex. In other words, at the centering point, there are equal effects from both the pressor and the depressor, indicating that the blood pressure is remaining constant. During the resting state, the value at point y was 100 mmHg. Fig. 2.1.3 shows that the centering point just represents the ultimate result of reflex regulation, i.e., the steady-state of blood pressure.

Since the baroreflex curve equation mentioned above was coined in the 1970s, it has been proved and refined time and time again by various types of physiological

世界图书出版西安有限公司

research for nearly half a century[1]. However, one particular question remained that was probably initially overlooked by physiologists: why is the centering point (the steady 100 mmHg of MAP maintained) situated exactly at the intersection between the diagonal of Cartesian coordinate system $y=x$ and the sigmoid stimulus-response curve $y=F(x)$(Fig. 2.1.4) Actually, this question can be easily answered by studying the results of repeated reflexes.

It turns out that when the actual MAP value (x) (equivalent to carotid sinus pressure under the physiological state) deviates and reaches a level that is much higher than 100 mmHg (Fig. 2.1.4), the body immediately alters the cardiac output and the peripheral vascular resistance to reduce the blood pressure. According to the sigmoid stimulus-response curve $y = F(x)$, when the blood pressure falls significantly below 100 mmHg due to certain postural changes, the carotid sinus immediately senses those new changes, and promptly elevates the blood pressure in response to the changes. The changes in the blood pressure are being continuously monitored by the carotid sinus, so that any further changes will be reset according to the stimulus-response curve, etc. This regulatory process can be observed where the values of x follow along the arrows in Fig. 2.1.4 like the trajectory of a cobweb, tracing back to exactly where the diagonal ($y=x$) and the curve $y=F(x)$ intersects. In other words, the intersection (near 100 mmHg) is like an attractor[2] , regardless of the values of x at the start, eventually, they are all

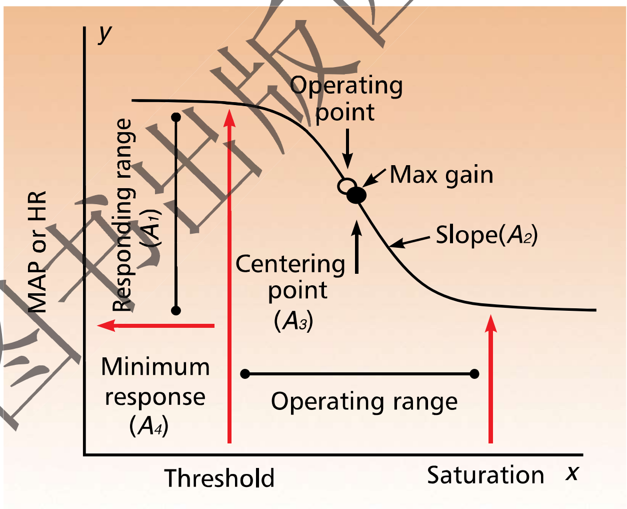

Fig. 2.1.3 The schematic model of the carotid baroreflex (CBR) functional curve and its operational parameters

世界图书出版西安有限公司

[1] Raven, P.B., et al., "Arterial Baroreflex Resetting During Exercise: a Current Perspective", Exp Physiol, Vol.91(2006); Chen, S. and Shi, X., "Re-Parameterization the Logistic Model in Assessing Changes in the Baroreceptor Reflex", *Neuroscience & Medicine*, Vol.2(2011).

[2] Attractor is a concept in Calculus and System Science that can be defined as having a tendency to develop toward a steady-state, such state is called an attractor. Attractors can be further categorized into non-strange and strange types. A pendulum system is a good example of a system that may be described as having a non-strange attractor, which causes the system to gradually move toward a steady-state of decreasing motion. There are three known types of non-strange attractors: the fix point (balance) attractor, the limit cycle (periodic motion) attractor, and the torus (quasiperiodic motion) attractor. Any other attractors besides non-strange attractors are considered strange attractors, which show the non-periodic and disordered system states in the chaotic system, such as the weather system.

attracted to the intersection of the diagonal lines (y=x) and y=F(x) according to the cobweb model. This process of attraction is the macroscopic expression of the acute blood pressure regulation.

This regulatory process can be expressed through mathematical language. The box in Fig. 2.1.5 represents the function y=F(x) (the causal relationship between stimuli and effects in the regulatory mechanism), i.e. when the carotid sinus detects the carotid pressure (x_1), the body immediately reacts to reset the systemic blood pressure (y_1) according to the function y=F(x) and the value of x. This new systemic blood pressure (y_1) is again quickly detected by the carotid sinus baroreceptor as x_2, which again resets the new systemic blood pressure (y_2) according to function y=F(x) and the new value x_2. In other words, the output of the box value y_1 at the first moment is also the input to the box value x_2 at the subsequent moment. As a result, the relationship between x and y becomes a self-coupling regulatory system as shown in Fig. 2.1.6. The term "self-coupling" means that the system output (specified by the input) is subsequently inputted at the next moment. Even though it is not yet clear what subsystems constitute the self-coupling system of Fig. 2.1.6, and what is the mechanism of their interaction or its mathematical expression, the result is crystal clear, i.e., the initial deviation of MAP can be corrected and quickly adjusted to be near 100 mmHg. Indeed, it is the end-result of the initial deviation of blood pressure being repeatedly processed in the self-coupling system.

Now we can comprehend why there are several different cases during the changes of the blood pressure in Fig. 2.1.2. The first and third cases were observed among the normal subjects because the slope of the reflection curve near the centering point is <

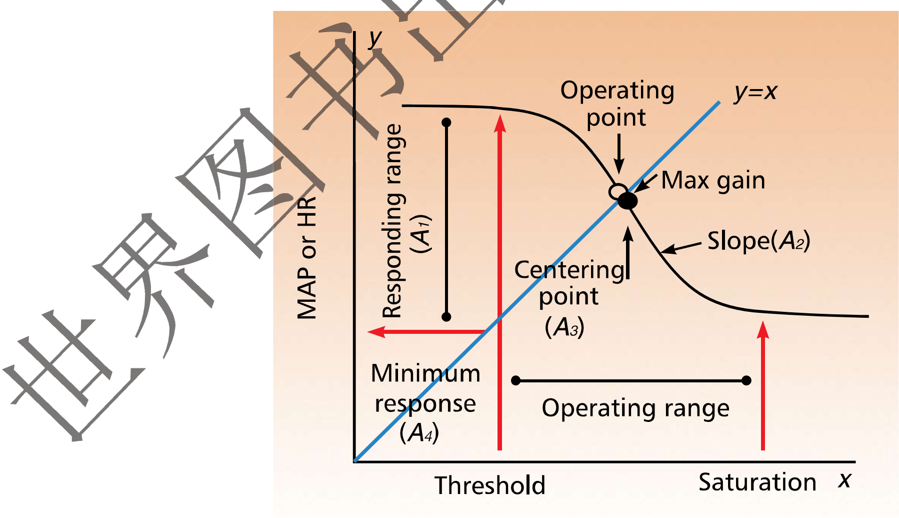

Fig.2.1.4 Reproduced from the schematic model of the carotid baroreflex (CBR) functional curve and its operational parameters (permission requested)[1]

[1] In fact, the curve in Fig. 2.1.5 is a product of editing, namely resulted from a selected A2 value of formula y=A_4+A_1/[1+exp{A_2(x-A_3)}]. If using the curve of Fig. 2.1.4 for self-recurrence, the result will show BP periodic oscillations or unstable BP, i.e., the intersection of the curve and y = x is not an attractor. Only the slope of the curve at the centralized point is < -1, and then the centralized point is the attractor.

世界图书出版西安有限公司

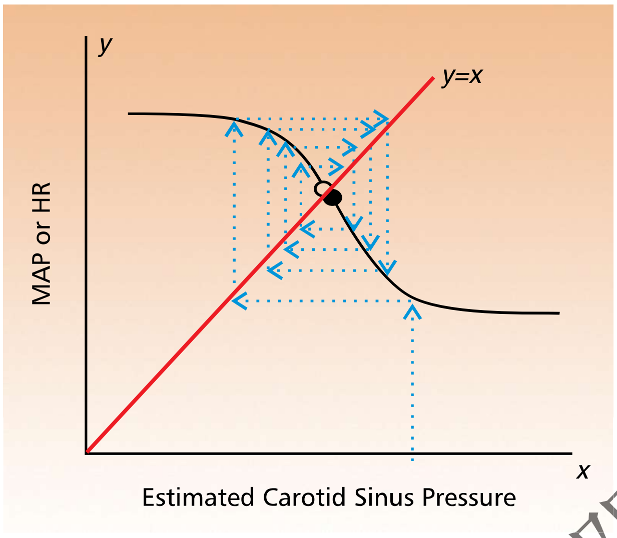

Fig. 2.1.5 The Self-coupling Representation of Baroreflex

-1. The fourth case refered to a situation that the slope of the reflection curve equals to -1, and the second case referred to a situation that the reflection curve had shifted. The above analysis implied that the body's extremely complex feedback regulatory mechanisms to maintain homeostasis of physiological variables could actually be expressed as self-coupling systems of the physiological curve. The result of overall regulation can be repeatedly calculated with the self-iterations of the physiological curve. The cobweb model of Fig. 2.1.5 illustrates such regulatory process.

Note that the feedback regulation in cybernetics is closely associated with two basic concepts: set point and goal discrepancy. The set point represents the goal of homeostasis. The goal discrepancy is the difference between the momentary status of the system and the goal value at a certain time. The regulatory mechanism to reduce the goal discrepancy is called negative feedback, while the regulatory mechanism to increase the goal discrepancy is called positive feedback. In the presupposition of cybernetics, any maintenance of homeostasis is impossible without set point and goal discrepancy. However, in the above analysis of self-coupling systems, there is no need to preset the set point and goal discrepancy. Therefore, we believe that self-coupling is a more basic concept than the feedback, because the feedback regulation is merely a special form of self-coupling system. The relationship between self-coupling and feedback will be discussed in detail in Chapter V. However, it is clear that self-coupling can exist wherever there is feedback. Therefore, in physiology, the premise of homeostasis should be self-coupling system, instead of the feedback regulation.

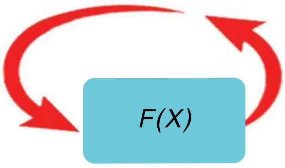

Fig.2.1.6 Mathematic Expression of Self-coupling Regulatory System

This modification of fundamental concept from self-coupling to feedback is extremely important due to the following reasons. First, the set point in the feedback regulation

世界图书出版西安有限公司

usually will be difficult to alter once it has been determined. If the steady-state value is presumed to be the set point, it will be impossible to understand the comprehensive causes for the deviation of the steady-state; especially when the deviated value could, on occasion, reach a number that's far greater than the normal value. In this case, there is no way to transit to systems pathology from systems physiology. Second, in physiology, it is known that functions of various subsystems composited into the human body can be expressed with corresponding physiological curves. Once a steady-state is formed by the self-coupling process, the link between steady-state and the function of its regulatory mechanism is established immediately.

The natural laws of physiology are based on the principles of physics, chemistry and systems biology. However, laws of physiology are not equivalent to those of physics, chemistry and systems biology, which consist of various physiological curves. As the human body is categorized into many smaller sub-systems, the relations between these subsystems are becoming increasingly complex, with many physiological curves of subsystems remaining unknown. However, due to the fact that each subsystem's function depends on their own structures and conditions, certain physiological curves of subsystems are always present, such as the above-mentioned stimulus-response curve of MAP where the rapid regulation is based on function $y=F(x)$ and the systemic MAP (y) is reset by the carotid sinus pressure (x). It can be said that physiology is the discovery of various physiological curves through experiments, as well as the science of studying their mechanisms and reciprocal relationships. By linking these physiological curves to the regulatory mechanisms of homeostases, the self-coupling analysis of the system theory can bridge the gap between physiology and pathology.

Thus, the following two important conclusions are attained. First, although the complex feedback mechanisms that maintain various types of homeostasis in the body are yet to be fully understood, they can be uniformly expressed as mathematical function of the self-coupling systems. The homeostatic values can be calculated by multiple self-iterations of the physiological functions, and the homeostatic points are the attractors of the self-coupling systems. Second, based on the first conclusion, the structural stability of the feedback system can be defined, and its impact on the steady-state can be analyzed, as well as the relationship between the structural stability and the diseased state (when the steady-state deviates from the normal values) can be found.

2.2 The Regulatory Mechanism in the Vicinity of Normal State (Origin Point)

According to the statement: "The normal steady-state value of MAP (100 mmHg) is at the intersection of the diagonal lines ($y=x$) and $y=F(x)$, which represents the relationship between stimulus-response curve of carotid sinus pressure and the post-regulation MAP ", the normal steady-state value of blood pressure satisfies the following equations:

$$x = y$$

世界图书出版西安有限公司

$$y = F(x)$$

Where $F(x)$ is the normal physiological curve

Namely $x=F(x)$ Equation 2.2.1

Equation 2.2.1 is the eigenequation with its solution being the eigenvalue of the self-coupling system. Obviously, whether the eigenstate is the normal values of the steady-state depends on whether it is the attractor of the self-coupling system. To determine this, the initial value x must be substituted into the function as an iterative calculation to see if the result converges to a certain point. When the convergence occurs, the solution of the eigenequation equals to the normal steady-state value.

Both terms of "eigenequation" and "eigenstate" come from quantum mechanics. They are used here for the reason that variable x can also be a vector, that is, it has several components of vector. In other words, when x is homeostatic, various components of x are also homeostatic. At that time, the F value in $x=F(x)$ is no longer a function, but a linear transformation of the vector, which can also be seen as a matrix. According to the above discussion, the meanings of eigenequation and eigenstate can both be defined as the invariance of the vector under linear transformation, which is exactly the definition of eigenstate in quantum mechanics. When x is not a scalar (a variable) but a vector (several variables), solving an eigenequation becomes more challenging, similar to those mathematical calculations of quantum mechanics [1]. But no matter how complex it is, the homeostatic process remains the same as the above definition, which is iterative transformation of the state vector. When it converges to a specific vector, such vector as the solution of eigenequation, is the steady-state, and it must also be an attractor.

In other words, when discussing the basic axioms of systems medicine in later sections, even though the state variable x is a vector in certain circumstances, it is still treated as scalar rather than vector for simplicity. Generally, the attained conclusion when x is treated as a scalar is the same for x as a vector in most cases. However, do note that all conclusions drawn from this book are only approximate. If some of these conclusions are deemed inconsistent with the pathophysiological facts, then they are probably caused by the simplification of x. Because for more accurate and rigorous analysis, such x must be treated as vectors instead of scalars for simplification. However, even with such simplification, the basic framework of systems medicine is still intact, and remains different from the medicine as we know today.

Once the state variable x is not a vector, there is a simpler way to determine if the eigenvalue is homeostatic or not. Let's look at physiological curve $y=F(x)$ as shown in Fig. 2.2.1, where $F(x)$ is treated as a line near the intersection of the curve and the diagonal $x=y$. If the intersection of physiological curve $y=F(x)$ and the diagonal $x=y$

[1] It must be noted that even though x is a vector, the method to calculate its eigenstates are different from those of Quantum Mechanics. In the intrinsic equation of Quantum Mechanics, observable quantities are the eigenvalues of the eigenstates, rather than the eigenstates themselves. In Systems Medicine, the eigenstates are physiological states that correspond directly to homeostatic values.

世界图书出版西安有限公司

is defined as the origin (x=0, y=0) in Fig. 2.2.1 [1], the function of physiological curve $y=F(x)$ is a straight line with a slope k. The stimulus-response curve as shown in Fig. 2.1.3 is simplified to the self-coupling system, in which the output and the input have a linear relationship as shown in Fig. 2.2.2.

Now, assuming that there is an output at the moment (t+1) when an input is provided at the moment t[2], namely $y(t+1)=kx(t)$. Since the system is self-coupling, the output in turn becomes the subsequent input, which is $x(t+1)=y(t+1)$. Thus,

$$x(t+n)=y(t+n)=kx(t+n-1)=k\cdot kx(t+n-2)\cdots=k\cdot k\cdot k\cdots kx(t).$$

According to $x(t+n)= k^n\cdot x(t)$, as long as the absolute value of k is < 1 ($-1 < k < 1$), when n approaches infinity, k^n approaches 0. At that time, no matter what value $x(t)$ is, the value of x ($t+n$) will eventually approach 0. The smaller the absolute value of the k, the faster the k^n approaches 0, which means the stronger the regulatory function of the system. This process of trending to 0 is the deviation of the steady-state being attracted to the origin (x=0, y=0) along the track as shown in the cobweb model of Fig. 2.2.1. For the system to reach the so-called steady-state, it means that x is no longer changing. No matter the value of $x(t)$, the y value of the self-coupling system approaching 0 is the solution of the eigenequation $x=kx$.

The above linear function is the expression of the regulatory mechanism to maintain the physiological curves near the steady-state. When proving the origin point 0 as the steady-state point, the premise is that the absolute value of k must be <1($-1<k<1$), so that the origin point 0 can be an attractor of a self-coupling system.

If the absolute value of k is >1($k<-1$ or $k>1$), according to $x(t+n)=k^n\cdot x(t)$, the value

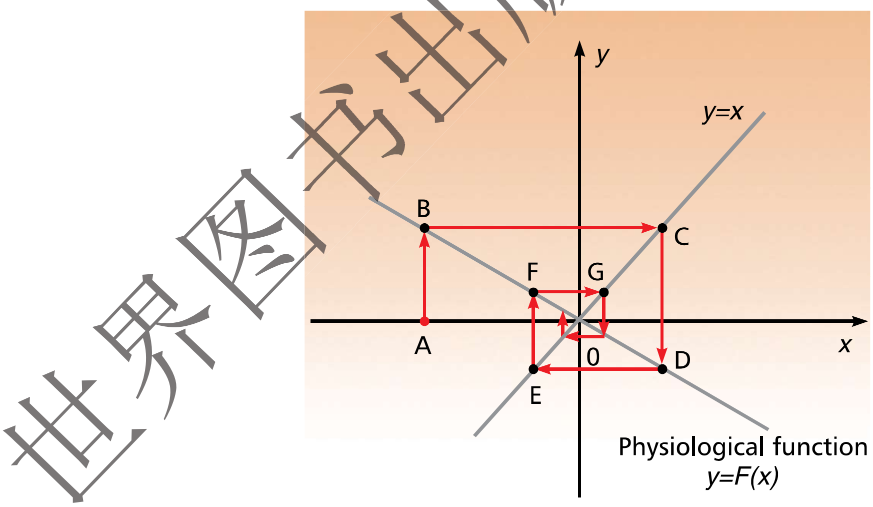

Fig. 2.2.1 Schematic Chart of Self-coupling Function Convergence

[1] All steady-state values are positive. In this book, the normal values of the steady-state is regarded as the origin of coordinate (both x and y values are 0), which is the result of translating the coordinate system. If the physiological function is written as $y = kx + b(0)$, the $s(0)$ of the steady-state is computed by the eigenequation $x = kx + b(0)$, where $b(0) > 0$. That is $s(0) = b(0)/1\text{-}k$. Obviously, $s(0) > 0$. If using s (0) as the origin, both x axis and y axis must be translated: x moves right to $s(0)$, y to $b(0)+kb(0)/1-k$. Therefore, s represents the difference between the eigenvalues and s(0).

[2] Here from t to t+1 indicates a single time interval, the intermittent regulations are used to express the continuous regulation. Obviously, in physiological regulation, the time interval is usually measured in seconds or minutes.

世界图书出版西安有限公司

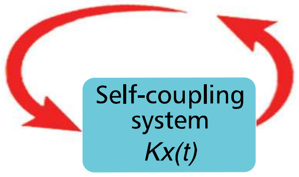

Fig. 2.2.2 A Simplified Schematic of Self-coupling System

of x will be ever increasing. Such regulatory process of the system is commonly known as positive feedback. When the value of k is equal to 1, the physiological curve overlaps with the diagonal line. For this case, although any number can be eigenvalue, they are merely fixed points or indifferent equilibriums [1], i.e., the system do not possess any regulatory capability to resist disturbance. When the value of k is equal to -1, k^n=1 or k^n=-1, the value of x oscillates between $x(t)$ and - $x(t)$. In other words, when the absolute value of k is near 1, the regulatory function of the system near the origin point 0 is gradually diminished. When the value of k is gradually reduced from 1, the regulation power of the system will be gradually enhanced and reaches the maximum with the value of k near 0. Such consequences also hold true for instances when the value of k goes from -1 to 0, in which 0 represents the strongest regulatory function, while -1 represents the regulatory function reaching the limit, because the system is then caught in the state of continuous oscillation [2], and becomes unstable, while the stability of the system is being gradually diminished. When the value of k is $<$ 1, a positive feedback occurs, and the steady-state is no longer attainable. As is well known, the above simple mathematical model can be used to express the feedback regulation, more importantly, this model can be used to study the structural stability of the regulatory mechanism and thus can be applied to systems medicine.

2.3 The Structural Stability of the Regulatory Mechanism

What is the structural stability of the regulatory mechanism? As mentioned previously, homeostasis refers to the mechanism that makes the system return to its original value after its status is being disturbed. Structural stability refers to the stability of the mechanism to maintain a steady-state, i.e. the system remains intact after being disturbed. Please do understand what is being disturbed here is actually the regulatory mechanism instead of the status of the system. More specifically, it is the pattern of the physiological curve.

Taking the rapid regulation of blood pressure mentioned at the beginning of this chapter for example. “The normal value of MAP was 100 mmHg” referred to human blood pressure value during the rest state. During exercise, in order to attain more

世界图书出版西安有限公司

[1] If the equilibrium state of an object does not change with the changing of time and position when influenced under external actions, or the equilibrium state deviates from the equilibrium position under minor disturbance but can recover at a new position, then this state is called "neutral equilibrium". For example, when a ball stops on a horizontal plane, or when a cone contacts its generatrix with a plane, a corresponding neutral balance occurs. When these objects are relocated to a new position, although they do not automatically restore the original position, they can remain at the new position without motion, and their height of the gravity center will be unchanged. Generally, as long as the gravity center of an object can be neither raised nor reduced by any minor movement, it must be in a state of neutral equilibrium.

[2] This concept was originally proposed by the Italian scientist Galileo. It means that in electromagnetic oscillations, if there is no energy loss, oscillations should last forever and the amplitudes should remain constant.

blood supply for motor organs (to ensure faster metabolism), normal MAP value was higher than 100 mmHg, with the blood pressure subject to change by the intensity of the exercise. However, the blood pressure during exercise remained homeostatic. For example, when a person suddenly tumbled and felled to the ground during exercise, the intracranial blood pressure of the person will quickly rise, triggering the blood pressure regulation to maintain the stability of the blood supply to the brain as shown in the self-coupling system of Fig. 2.2.2. In this case, the steady-state point of the blood pressure is still at the intersection of the stimulus-responses curve and the diagonal. The stimulus-responses curve of the motion status is different from that of the rest state, and it shifts upward along with the change of intensities of the exercise. The shift of stimulus-responses curve can be quite complex, an example of that shift is the change of A to D in Fig. 2.3.1, where the intersection value between the curve and the diagonal naturally become greater.

In the above case where the rising blood pressure was caused by exercise, the upward shift of the curve of the blood pressure regulatory mechanism (regulatory function) was simply a result of the demanding exercise and the rising homeostatic value of blood pressure was not a diseased status. However, if the blood pressure curve consistently shifts up and down or distorts during rest, it indicates that there is disturbance of the regulatory mechanism, resulting in stressed or diseased statuses of human body, etc. Therefore, one can use the transited or distorted curve that represents the regulatory mechanism to define its disturbances, as well as the structural stability of the system by its response to such disturbances. In all, if the steady-state of the regulatory mechanism can be defined by their ability to return to the normal values post-disturbance, then the structural stability of the system manifests as the changes of the entire system when the regulatory mechanism is disturbed. In this case, as the curve of the regulatory function is transited or distorted, the result must be the change of the intersection between the curve and the diagonal, that is, the steady-state value deviates from the normal values. At this point, if the system is not destroyed (i.e. the

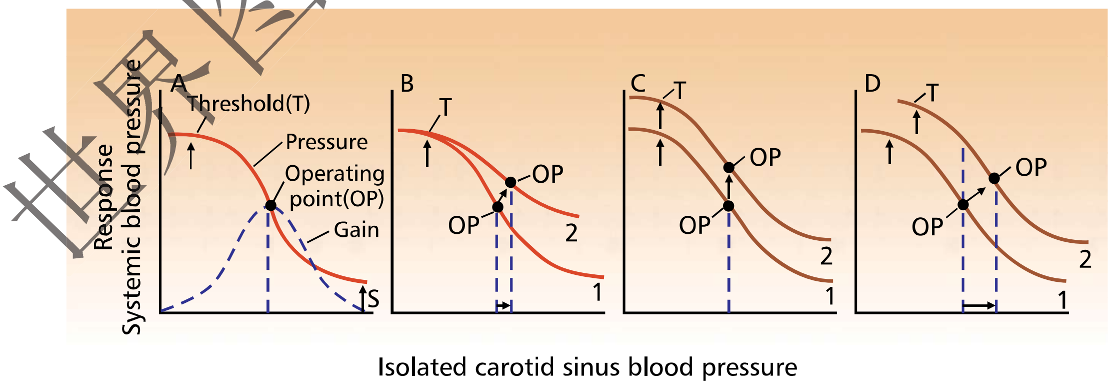

Fig. 2.3.1 The Resetting of Barorecepter from Rest to Exercise[1]

[1] Rowell, L.B., O'Leary, D.S., Kellog Jr., D.L., "Integration of Cardiovascular Control Systems in Dynamic Exercise", in Rowell, L.B., Shepherd, J. T.,ed., Handbook of physiology: a Critical, Comprehensive Presentation of Physiological knowledge and concepts, Oxford University Press, 1996.

世界图书出版西安有限公司

person is not dead), there must be at least the following three scenarios.

First, when the disturbance subsides, the physiological curve, which represents the regulatory mechanism, will slowly restore to the undisturbed status, that is, the system status will self-recover. In other words, similar to the maintenance of homeostasis, the disturbance of the regulatory mechanism can also be overcome by an advanced stabilization mechanism, so that the regulatory mechanism returns to its original, undisturbed status[1].

Second, even if the disturbance remains, or the system post-disturbance is not able to restore immediately, the regulatory ability of self-stabilization (stabilization mechanism) is not lost. Specifically speaking, the stabilization mechanism is represented by the self-coupling of the physiological curve, when the stabilization mechanism is disturbed, the curve becomes distorted, and the homeostatic value defined by the curve must deviate from the normal values. However, as long as the degree of the curve distortion does not cause death (i.e. the system is not destroyed), the system remains intact and continue to play its role. For example, the blood pressure in patients with hypertension is still homeostatic, only the value of their attractor is higher than the normal values.

Third, the disturbance is usually controllable. "Being controllable" does not allude that there must be a solution to eliminate the disturbance, but actually means that a minor shift in physiological curve does not lead to a significant deviation (mutation) of its homeostatic value. For example, though the homeostatic value of blood pressure may decrease or increase with postural changes or exercises, a healthy person doesn't easily go into shock or has the blood pressure, the value of which is far less or much greater than normal, resulting in a significant impact on other systems of the body. This viewpoint can be accurately expressed as the smaller the disturbance, the smaller the deviation of the homeostatic value.

For the first scenario, it simply means that human has the ability of self-healing, and the second scenario indicates the occurrence of the disease. Chapters IV, V, and VI of this book will show that the third scenario corresponds to the feasibility of the treatment. Accordingly, it is important to know why the homeostasis maintained by feedback regulation is called the basis of physiology, and why the study of structural stability is considered as the basis of pathology and therapeutics, as previously mentioned in Chapter I. In other words, reveal of a series of feedback mechanisms that maintain various homeostases of the human body allows us to understand why the life system can exist under various disturbances. By further understanding of the structural stability of the regulatory mechanism that maintain various homeostases, we can discover how regulatory mechanisms themselves are disturbed, how they still maintain regulatory ability post-disturbances, and even how disturbances are eliminated to attain the ultimate goal of self-healing. All of these viewpoints constitute the basis of pathophysiological researches and therapeutics.

[1] Generally, system theory considers the existence of homeostasis as the stability of the system, and the stability of the homeostatic mechanism is on a higher level than the stability of the system, which is called structural stability.

世界图书出版西安有限公司

2.4 The Shift of Homeostasis under Linear Approximation

In the previous sections, a homeostasis has been expressed as a self-coupling curve of physiological variable mathematically. The mathematical expression of homeostatic mechanism can also be applied to the study of the structural stability as below. If $a \cdot F(x)$ represents the disturbance of the original function $F(x)$ of the curve, a new function $F(x)+a \cdot F(x)$ will be the result post-disturbance. According to the definition of the structural stability of the system in the Sec 2.3, there are three scenarios.

First, due to a more advanced stabilization mechanism in place, the disturbance $a \cdot F(x)$ slowly disappears, which means the value of a finally becomes 0.

The second is that the eigenvalue of the function (the value of steady-state) changes, but it does not exceed the range required for the maintenance of life. The disturbed eigenvalue of a self-coupling system can be solved with the following eigenequation formula.

$$x=F(x)+a \cdot F(x) \qquad \text{Formula 2.4.1}$$

In the third scenario, when the value of a is sufficiently small and even near 0, the eigenvalues calculated by Formula 2.4.1 will be infinitely near the eigenvalues attained by Equation 2.2.1. In other words, the smaller the disturbances of function in the self-coupling system, the smaller the deviation of the steady-state from the normal values.

The above three scenarios constitute the basic framework of systems medicine. As long as the function of regulatory system is linear, the third scenario already encompasses the first two scenarios. In other words, when the function is only a straight line, its disturbance only manifests as its linear transition and slope changes, and its intersection with the diagonal is no longer the origin point 0, but point s (Fig. 2.4.1).

The s value can be derived through simple mathematics. Following the discussion in Sec. 2.2, under some disturbances, the regulatory function $y=kx$ near the origin becomes another linear function: $y=kx+ax+b$. The value of a represents any disturbance to the regulatory mechanism; the value of b represents any linear disturbance to the system (shift up and down). Both of them constitute the disturbance to the homeostatic mechanism.

A new steady-state, which satisfies the following formula, can be generated in the self-coupling system:

$$\begin{cases} y=kx+ax+b \\ y=x \end{cases} \qquad \text{Equation 2.4.2}$$

Accordingly, the eigenequation is $x=(k+a)x+b$. If the solution of the equation is rewritten as s (the deviation value of the steady-state), the following formula can be attained:

$$S=\frac{b}{1-(k+a)} \qquad \text{Formula 2.4.3}$$

世界图书出版西安有限公司

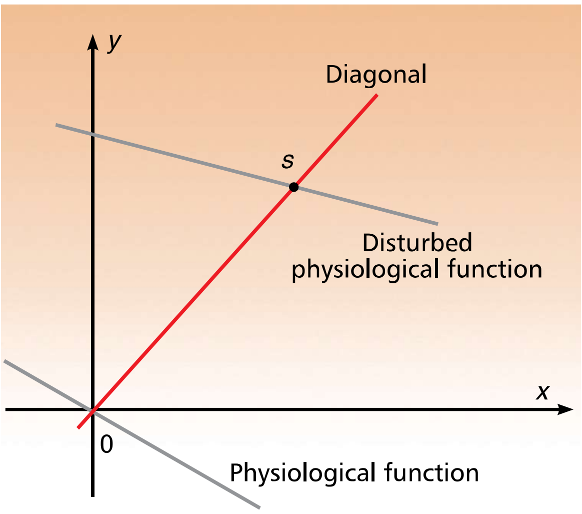

Fig.2.4.1 The Shift of Intersecting Point between the Diagonal and the Physiological Function Post-Disturbance

In the Formula 2.4.3, when the absolute value of (k+a) is<1, the smaller the absolute value of b, the smaller the value of s, which is also an attractor that represents a new steady-state value. When s approaches 0, the deviation of the steady-state value from the normal values post-disturbance will become less and less.

For non-linear systems, it is much more complex [1], but the self-coupling analysis is still applicable. Meantime, $F(x)$ is a non-linear function of x, and the above cobweb model can still be applied to judging whether the intersection of $F(x)$ curve and the diagonal is in the steady-state. Obviously, the number of eigenstates can be derived from the total number of possible solutions attained from $x=F(x)$. Although the non-linear self-coupling system is complex, a very useful theorem can be applied: it is impossible for both of the two adjacent intersections of the $F(x)$ curve and the diagonal line to be in steady-states. In order to determine which one is in steady-state, there is a method that expands $F(x)$ to a power series in the vicinity of each eigenvalue. When $-1 < F'(x_0) < 1$, it is in steady-state [2]. When $F'(x_0) = 1$, in which higher order derivatives are considered, there will be a steady-state without structural instability. When it is disturbed, the formula $s=b/[1-(k+a)]$ (Formula.2.4.3) will no longer be valid. This viewpoint is important for systems medicine, however, the controllability of medical intervention is often emphasized in therapeutics, and the linear relationship is the premise to controllability. This book, as an introduction to systems medicine, only

世界图书出版西安有限公司

[1] In systems medicine, the homeostatic physiological curves formed by self-coupling when its slope is near or equal to 1 at the steady-state point is also known as non-linear systems. Such researches warrant further study via Systems Medicine.

[2] For a non-linear self-coupling system, when its within the vicinity of x (0). it may behave like the linear system and is faced with three potential scenarios: (1) x (0) is the steady-state; (2) There is a periodical oscillation; (3) A type of vibration with increasing amplitude occurs. However, in regions far from x (0), the non-linear system may manifest other scenarios in addition to the above-mentioned. It was found that such behaviors of non-linear self-coupling system are very peculiar. In some cases, although the change of x value was predetermined, its fluctuations were random. This is the so-called chaos. D. R. Hofstadter knows the process of state variables approaching the steady-state value in the self-coupling system known as the "strange attractor." This term is quite descriptive, because the method of cobweb model has proved that the value of x will eventually reach 0 (the x (0) state), no matter the initial value of x. The steady-state of function in the self-coupling （The next turn）

discusses the linear simplification and accordingly proposes the basic framework of systems medicine. After the basic framework is established, we can further deal with the non-linear and other more complex situations, which belong to a more advanced study of systems medicine.

In the forthcoming Chapter Ⅲ, the method of self-coupling analysis applied in the acute blood pressure regulation will be applied to the entire scope of physiological and pathological studies to propose the axioms of systems medicine. A so-called axiom is a basic concept expressing the homeostasis and its structural stability from individual cases; it is also the premise of systems medicine and will be used in place of the prevailing causal analysis framework in conventional clinical medicine. As mentioned previously in Chapter I, the causal analysis framework currently applied to medicine was postulated and has numerous shortcomings or drawbacks. Now, it is time to proceed with an "out with the old, in with the new" approach and revamp the framework with systems medicine. This book will again show that the conclusions drawn from this new framework are far more accurate than the causal analysis.

世界图书出版西安有限公司

(On the ground) system seems to always be a center of attraction. Realistically speaking, the state variables of the system reach the steady-state values as if a small ball falls down to the valley bottom due to gravity. The location of the valley is exactly the steady-state point $x(0)$. A. Lyapunov was the first person to describe the steady-state with such vivid imaging. By studying the stability of differential equations, he proposed Lyapunov function $V = V(x, y)$, which was constructed by equations and can be thought as a curved surface (or hypersurface) of phase space $(x * y)$. If there is a valley in the surface of the potential function, the valley bottom represents the steady-state, and the size of the valley represents the sphere of influence surrounding the attraction center. When an eigenequation has the steady-state point $(x(0), y(0))$, the value of this point must be the minimum of $V(x, y)$. It can be determined from the equation that when $x \neq x(0)$, $y \neq y(0)$, there must be $dv / dt < 0$. That is whatever the state the system began with; as long as it is located within the valley specified by Lyapunov function, it will surely be attracted to the steady-state point. For the self-coupling system, a potential function similar to the Lyapunov function can also be introduced so that the corresponding steady-state point $x(0)$ is also expressed as a valley of the potential function, though the variation trajectory of the function specified by the cobweb model is discontinuous. As long as the self-coupling system is linear, it must also be structurally stable. However, for non-linear systems, it is not necessarily stable, which depends on whether the k is negative or positive at the vicinity of equilibrium point. When k is negative, the non-linear system is still structurally stable. When k is positive, it may be equal to 1. At this time, the system is structurally instable. Any small disturbance may separate the steady-state into two new steady-states. According to Chapter IV, a positive k means that the regulation of self-coupling system is constrained. Thus, an important conclusion can be drawn here: only the constrained regulation makes a steady-state with the structural instability.
